# Single nucleotide polymorphisms and cancer susceptibility

**DOI:** 10.18632/oncotarget.22372

**Published:** 2017-11-07

**Authors:** Na Deng, Heng Zhou, Hua Fan, Yuan Yuan

**Affiliations:** ^1^ Tumor Etiology and Screening Department of Cancer Institute and General Surgery, The First Affiliated Hospital of China Medical University, and Key Laboratory of Cancer Etiology and Prevention (China Medical University), Liaoning Provincial Education Department, Shenyang 110001, China; ^2^ Department of Hematology, The Fourth Affiliated Hospital of China Medical University, Shenyang 110001, China; ^3^ National Clinical Research Center for Digestive Diseases, Xi’an 110001, China

**Keywords:** single nucleotide polymorphism, genetic, epigenetic, susceptibility, cancer

## Abstract

A large number of genes associated with various cancer types contain single nucleotide polymorphisms (SNPs). SNPs are located in gene promoters, exons, introns as well as 5'- and 3'- untranslated regions (UTRs) and affect gene expression by different mechanisms. These mechanisms depend on the role of the genetic elements in which the individual SNPs are located. Moreover, alterations in epigenetic regulation due to gene polymorphisms add to the complexity underlying cancer susceptibility related to SNPs. In this systematic review, we discuss the various genetic and epigenetic mechanisms involved in determining cancer susceptibility related to various SNPs located in different genetic elements. We also discuss the diagnostic potential of these SNPs and the focus for future studies.

## INTRODUCTION

Single nucleotide polymorphisms (SNPs) are one of the most common types of genetic variations in the human genome. SNPs in genes that regulate DNA mismatch repair, cell cycle regulation, metabolism and immunity are associated with genetic susceptibility to cancer [1–12]. Understanding the mechanisms underlying the effects of SNPs that result in cancer susceptibility is critical to understanding the molecular pathogenesis of various cancers. From a clinical perspective, SNPs are potential diagnostic and therapeutic biomarkers in many cancer types.

SNPs are located in different regions of genes such as promoters, exons, introns as well as 5′- and 3′ UTRs. Therefore, alterations in gene expression and their effect on cancer susceptibility vary depending on the location of the SNPs. The promoter region SNPs affect gene expression by altering promoter activity, transcription-factor binding, DNA methylation and histone modifications [13–20]. The exonal SNPs affect cancer susceptibility by suppressing gene transcription and translation [21–23]. SNPs in intron regions generate splice variants of transcripts and promote or disrupt binding and function of long non-coding RNAs (lncRNAs) [24–26]. SNPs in the 5′-UTR affect translation, whereas SNPs in the 3′-UTR affect microRNA (miRNA) binding [9, 27–29]. SNPs in regions that are located far from the actual genes reduce or enhance gene transcription through long-range cis effects [30]. In this systematic review, we discuss the genetic and epigenetic mechanisms that underlie the SNP-related cancer susceptibility and the potential utility of SNPs as cancer biomarkers.

## PROMOTER REGION SNPs AND CANCER SUSCEPTIBILITY

**Figure 1:**
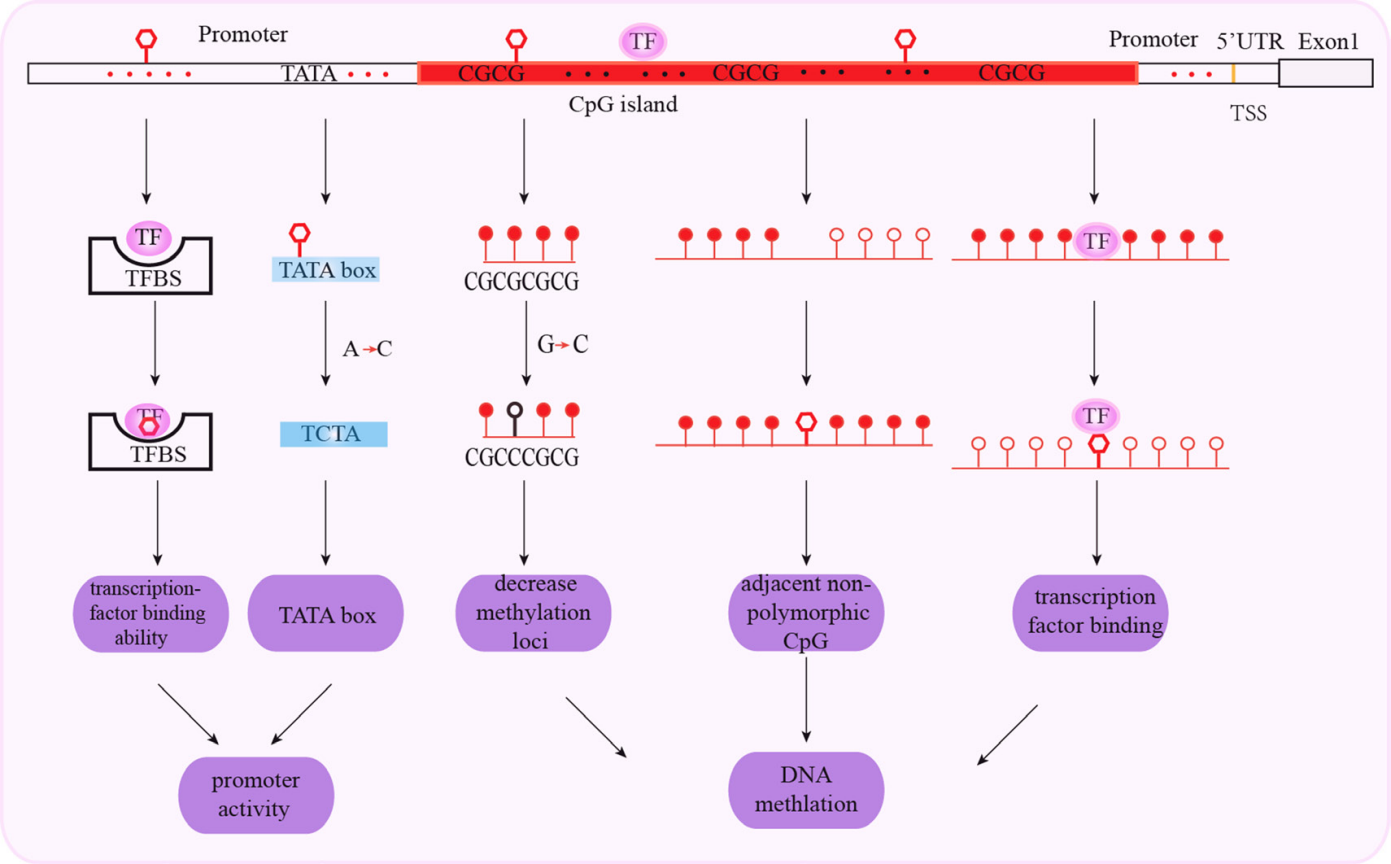
Schematic representation of mechanisms associated with promoter SNPs and cancer susceptibility SNPs in transcription factor binding sites affect transcription factor binding to the gene promoter. SNPs in the TATA box affect promoter activity with A to C substitutions decreasing the number of the TATA boxes. SNPs in the CpG islands decrease methylation, affecting adjacent non-polymorphic CpG and transcription factor binding. The red triangle represents SNP; red arrows show substitutions of SNPs; red hollow circle represents unmethylated loci; red solid circle represents methylated loci.

The promoter region regulates the initiation and rate of gene transcription through cis-acting elements and trans-acting factors. Promoter-related polymorphisms affect transcription factor binding that alter promoter activity, gene transcription, mRNA stability and translation. Subsequently, these effects alter protein levels that potentially determine the individual's susceptibility to diseases including cancer. Moreover, polymorphisms in the promoter regions also affect cancer susceptibility by altering epigenetic mechanisms such as DNA methylation and histone modifications (Figure 1).

### Promoter region SNPs affect promoter activity

#### Effect of promoter region SNPs on TATA box function

Polymorphisms in the TATA box inhibit promoter activity and decrease genetic transcription because it is part of the core promoter. For example, a point mutation (A > C) at position -27 in the putative TATA box of the *EDH17B2* gene decreases promoter activity [31].

#### Effect of promoter region SNPs on transcription factor binding

The promoter region contains numerous binding sites for transcription factors that regulate gene transcription. Polymorphisms in the promoter region decrease the transcription of target genes by inhibiting the binding of the transcription factors to the promoter. The rs16260 (-160C > A) and rs5030625 (-347G-> GA) in the *CDH1* promoter reduce transcriptional activity to different degrees [32, 33]. The rs16260(-160C > A) SNP decreases gene transcription by inhibiting transcription factor binding at the *CDH1* promoter and promotes tumorigenesis, including prostate,breast, colon and pancreatic cancers, etc [32]. The *CDH1* rs5030625 (-347G->GA) decreases CDH1 expression by 10-times (P < 0.001) via inhibiting transcription factor binding and correlates with increased familial gastric cancer susceptibility [33]. Conversely, *APE1* rs1760944(-656T > G) is associated with increased transcription factor binding and higher cervical cancer risk [34].

Promoter region SNPs modulate binding between transcription factor and promoter based on the number of binding sites for transcription factors such as SP1, c-Myb, E2F1, Ets, and GATA-1. For example, the rs2596538 in the *MICA* promoter region increases the risk of hepatitis C virus-associated liver cancer (OR = 1.34) with the transcription factors binding to the G allele and not the A allele [35]. The rs689466 (-1195G > A) SNP in the promoter region of the *COX-2* gene generates a new c-Myb binding site, thereby enhancing *COX-2* expression and increasing the risk of oesophageal cancer by 1.72-fold (95% CI, 1.35–2.20) [36]. A T→G substitution rs2279744 in the promoter region of *MDM2* increases transcriptional activity by enhancing the binding between the promoter and MDM2 transcripts; MDM2 overexpression mediated by cyclin D1 promotes tumorigenesis [37]. The rs1799750 -1607 1G→2G polymorphism in the *MMP-1* promoter generates a Ets binding site that increases *MMP-1* expression [38]. The C→T transitions at -1306 and -735 in the *MMP-2* promoter eliminate a Sp1-binding site (CCACC box), thereby decreasing MMP-2 transcription and increasing the risk of esophageal squamous cell carcinoma (OR = 6.53; 95% CI = 2.78–15.33) [39]. The rs8179090 (-418G > C) in the *TIMP-2* promoter eliminates the Sp1-binding site and decreases TIMP-2 expression [40].

SNPs in cis-acting elements such as GATA-1 transcription factor binding sites enhance the promoter activity of the survivin gene in breast cancer patients. For example, The G→A transitions at -235 SNP in the promoter of survivin gene generates a second GATA-1 binding site in its promoter region, thereby increasing survivin expression in breast cancer tissues [41].

### Promoter region SNPs impact epigenetic mechanisms

#### Effect of promoter region SNPs on DNA methylation

DNA methylation occurs primarily in the CpG islands of the promoter region. Therefore, SNPs in the promoter region can alter DNA methylation status and profoundly impact gene expression. The SNPs associated with DNA methylation of CpG loci are referred to as methylation SNPs [15, 42–44]. SNPs in genes rich in G and C bases are frequently mutated in many human diseases. SNPs in the promoter region alter the number of CpG dinucleotides in a CpG island, leading to changes in methylation, histone acetylation, chromatin modification, and gene silencing [45].

#### Promoter region SNPs alter the number of methylation loci, thus changing gene expression and increasing the risk of cancer

Some promoter region SNPs alter methylation in an allele-specific manner. Genome wide association studies (GWAS) showed that 38 SNPs in 12 CpG loci correlated with changes in methylation and expression of 10 genes (*IRF6, TSPYL5, CRIM1, CHL1, DDT, PIGC, TMOD1, ZNF266, BDKRB2, GSTT1*) [46]. Zhang *et al.* reported that the CHEK2 rs2236141 (-48 G > A) variant was associated with lower lung cancer risk (adjusted OR = 0.73) because it eliminated a methylation locus, thereby relieving transcriptional repression [47]. The EZH2 rs6950683 C allele showed reduced OSCC risk compared to the wildtype T allele because it was methylated and resulted in lower EZH2 expression [48].

Furthermore, CpG SNPs affect many non-imprinted autosomal genes in normal human tissues by allele-specific DNA methylation (ASM), allele-specific gene expression (ASE) and allele-specific transcription factor binding (ASTF) [49]. SNPs in the promoter region modulate cytosine methylation of adjacent non-polymorphic CpG sites by increasing or decreasing the methylated loci. Methylation is more effective in regions of high CpG density or regions with abundant CpG SNPs since they promote effective binding of methylating enzymes and related factors.

#### Promoter region SNPs affect transcription factor binding that protects CpG islands

DNA-protein interactions in the promoter region can influence the methylation status and gene transcription. For example, transcription factors such as SP1 and CTCF influence methylation on CpG islands [49]. In sporadic breast tumors, methylation status was altered by mutations in the SP1 and CTCF binding sites in the promoter, which correlated with *BRCA1* downregulation [50]. SNPs in the Sp1-binding domain of RIL which is a frequent methylation target in cancer prevent SP-1 binding and increase CpG methylation. Boumber *et al.* showed that SNPs influence time-dependent gene silencing by altering methylation levels [51].

#### Promoter region SNPs alter DNA methylation by activating methylation-related enzymes

Polymorphisms in the promoter region of DNA methyltransferase (*DNMT*), methylene tetrahydrofolate reductase (*MTHFR*) and methionine synthase (*MS*) inhibit DNA synthesis and promote abnormal DNA methylation. Ogino *et al.* identified a common MGMT promoter SNP, rs16906252 (-56C > T), which recruited DNA methyltransferases resulting in loss of MGMT expression in colorectal tumors [52].

#### Promoter region SNPs affects histone modification

Polymorphisms in non-coding regulatory sequences alter histone modifications such as acetylation, methylation, phosphorylation, ubiquitination, and glycosylation, which affect transcriptional rates. Promoter SNPs located in transcription factor binding sites are associated with regional histone modifications. SNPs in distant regulatory regions also regulate gene expression [53]. For example, rs1800896 (A-1082G) GG genotype has been found to be associated with high levels of IL-10 production [54]. Lipopolysaccharide (LPS) treatment increased acetylation of H4 histones and methylation of histone H3 in lymphoblastoid cells with GG genotype than cells with AA genotype. Conversely, lymphoblastoid cells with AA genotype showed increased histone H3 acetylation than cells with GG genotype. In unstimulated lymphoblastoid cells, cells with GG genotype showed higher levels of acetylation and methylation of histone H3 than cells with AA genotype, whereas cells with AA genotype showed higher levels of acetylation of histone H4 [55].

## EXONAL SNPS AND CANCER SUSCEPTIBILITY

**Figure 2:**
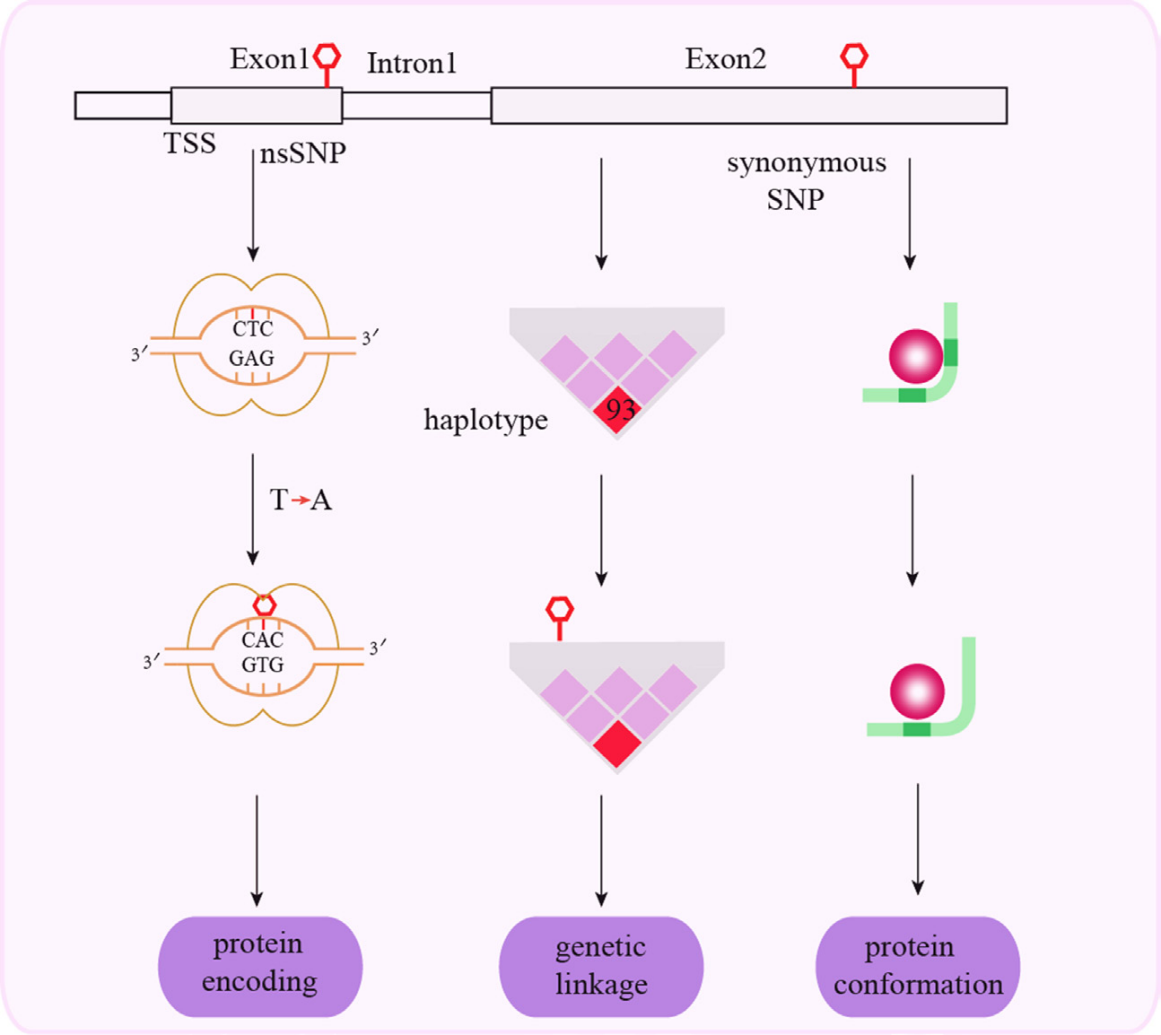
Schematic representation of mechanisms associated with exonal SNPs and cancer susceptibility Non-synonymous exonal cSNPs change the amino acid sequence of the encoded protein. Synonymous exonal cSNPs change protein conformation and function via genetic linkage.

SNPs in exons are classified as non-synonymous and synonymous coding SNPs (cSNPs) based on their ability to replace the encoded amino acid. Exonal SNPs generally influence cancer susceptibility by genetic mechanisms (Figure 2).

### Non-synonymous cSNPs change protein structure and function

Non-synonymous cSNPs result in amino acid substitution that can affect protein function. Changes in the first two bases of a codon result in amino acid changes in most instances. Alterations in the amino acid sequence can alter the secondary structure of the protein by increasing or decreasing hydrogen bonding and phosphorylation, which affects protein interactions and functions. As a consequence, these changes alter cell signaling pathways as well as levels of oncogenic and tumor suppressor proteins. More than 13,000 known SNPs are in exons of various genes, of which 58% are non-synonymous cSNPs [56]. Non-synonymous SNPs influence cancer susceptibility due to changes in the structure and function of the encoded proteins. For example, a non-synonymous cSNP in the epidermal growth factor gene (*EGFR*) eliminates the tyrosine kinase domain (TKD), which is targeted by small-molecule tyrosine kinase inhibitors (TKIs) such as gefitinib and erlotinib. Erlotinib forms a single hydrogen bond with Met 769 in the EGFR, whereas gefitinib forms two hydrogen bonds with Gly 772. Gefitinib shows higher affinity with five polymorphic EGFR proteins than the wild-type EGFR. Polymorphisms in the EGFR-TKD lead to structural changes which increase protein activity and sensitivity to TKIs [57]. The *NAT2* (N-acetyltransferase 2) gene polymorphism rs752955201 results in substitution of valine by a bulkier isoleucine which decreases its affinity for NAT2 substrates and increases its affinity for NAT1 substrates [58].

A non-synonymous cSNP that changes amino acid sequence of the protein-protein interface (PPI) can alter protein interactions, affect stability, and alter post-translational modifications [59]. The Leu858Arg mutation increases the ability of EGFR to form dimers and is associated with cell proliferation [60]. Gain-of-function mutations in tumor suppressor gene p53 promote tumorigenesis [61]. Coincidently, many unclassified mutations are found at protein-protein interfaces [62].

### Synonymous cSNPs alter protein structure and function indirectly

Synonymous cSNPs do not alter the amino acid sequence of the encoded protein. In most cases, the nucleotide change is associated with substitution in the third base in a codon. Since the amino acid sequence of these proteins is the same as wild type, they were previously not considered important. However, recent studies show that synonymous cSNPs affect gene function and expression by changing the expression of neighbouring genes. Many studies have demonstrated that synonymous mutations alter the structure, function, and expression of proteins.

### Synonymous cSNPs change secondary structural conformation of mRNAs and proteins

Synonymous cSNPs form different haplotype SNPs that modulate the stability of mRNA secondary structure (local stem-ring structure), thereby reducing enzyme function. For example, two synonymous cSNPs and one non-synonymous cSNP in the Catechol-O-Methyltransferase (*COMT*) gene form different haplotype SNPs. The major COMT haplotypes show variations in the local stem-loop structures of messenger RNA. The mRNAs with stable secondary structures are associated with low COMT expression and activity [63]. A synonymous SNP in multidrug transporter (*MDR1*) gene alters the expression of the critical drug transport protein, P-glycoprotein. This affects its expression and function, thereby impacting drug resistance. SNPs in *MDR1*, namely, 1236C→T, 3435C→T, and 2677G→T SNPs occur in 31-49% of Chinese, Malaysian, and Indian populations [64]. Strong linkage disequilibrium among these three SNPs generates a haplotype which decreases the function of the MDR1 protein with subtle changes in the conformation of its ATP-binding site. The T1236C and C3435T *MDR1* SNPs have the potential to influence non-synonymous SNPs. The C3435T polymorphism affects co-translational folding and insertion of P-glycoprotein into the membrane, thereby altering the structure of substrate-inhibitor interaction sites [65]. The three SNPs represent rare wild-type codons, which are associated with differences in translational rate and termination of translation [66]. The resulting differences in drug-substrate binding represent a possible mechanism by which synonymous SNPs regulate non-synonymous SNPs and result in diversity of clinical responses [67].

Synonymous polymorphisms affect messenger RNA splicing, stability, and structure as well as protein folding. These changes significantly affect function of proteins resulting in changes in cellular response to therapeutic targets, which explains differential responses of individual patients to medications [68]. For example, rs74090726 synonymous polymorphic variation in MCAD exon 5 inactivates the exonal splicing enhancer (ESE) causes exon skipping thus leading to loss of a functional protein and to MCAD deficiency [69]. The synonymous SNPs can lead to diverse diseases by changing microRNA-mediated gene regulation and further altering the gene expression based on bioinformatics analysis [70].

### Synonymous cSNPs alter translational rates via genetic linkage

Synonymous cSNPs can also accelerate or decelerate the speed at which the ribosome moves along the mRNA, thus changing the dynamics of translation, and the subsequent protein structure and function. They may also result in different mRNA secondary structures and protein secondary structures such as α helix β folding [71]. Moreover, codon usage in synonymous codon families is not random and base-structure preference is associated with the position of the second base in the codon [72]. In some cases, the base-structure preference is related to the third base [73].

## INTRONAL SNPs AND CANCER SUSCEPTIBILITY

**Figure 3:**
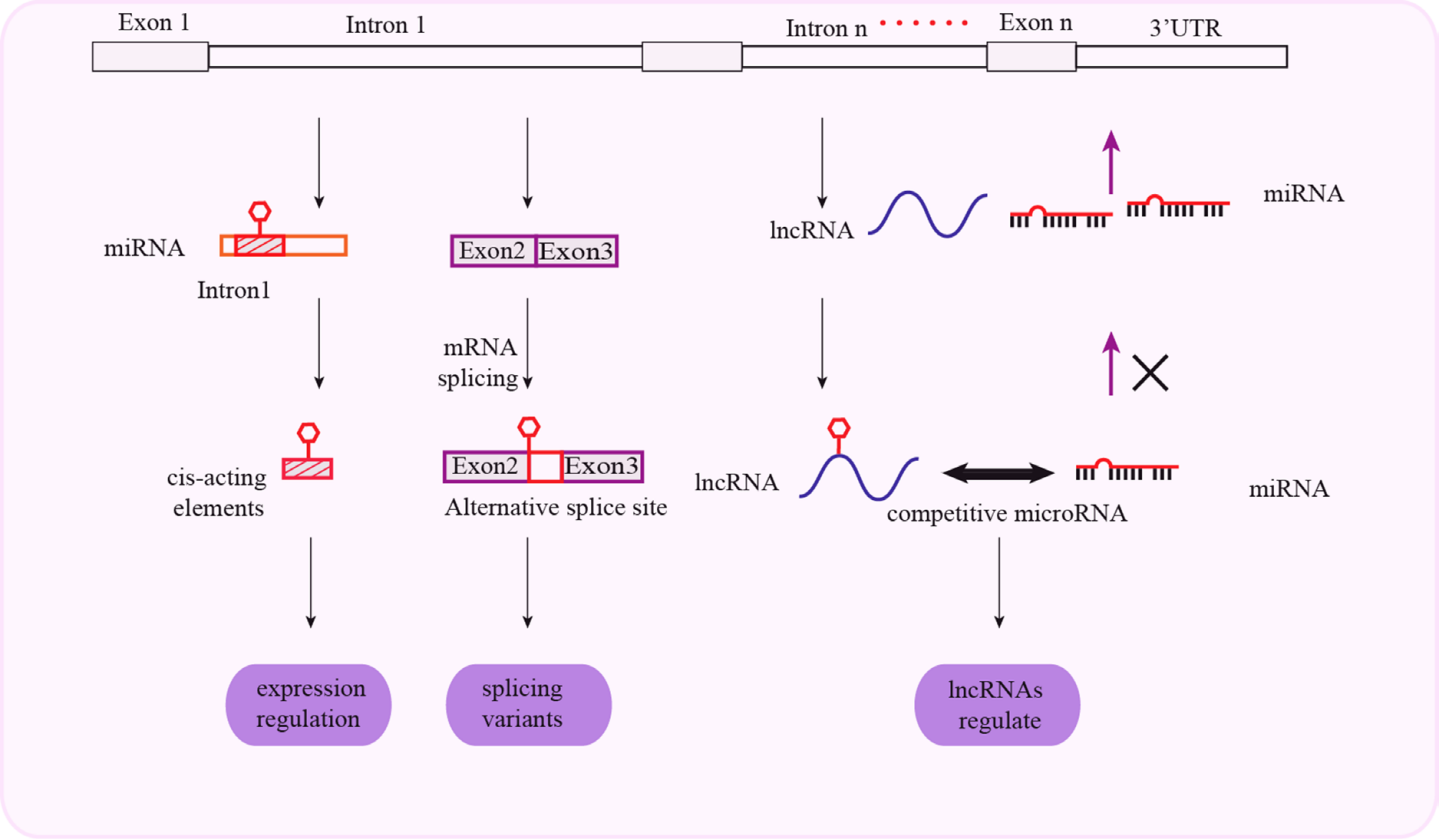
Schematic representation of mechanisms associated with intronal SNPs and cancer susceptibility Intronal SNPs influence gene expression through cis-acting regulatory elements. Intronal SNPs influence protein synthesis by mRNA splicing and regulation of lncRNA function.

Introns are involved in regulating tissue-specific gene expression, mRNA transcription, and translation. They contain enhancers or other cis-elements that promote transcription initiation or elongation. Intron splicing increases mRNA stability in the nucleus. Introns are also involved in alternative splicing and genome imprinting. Functional SNPs in introns are sometimes linked to SNPs of nearby genes, affecting their mRNA splicing and lncRNA binding. This results in variations in the sequence and function of the mature proteins.. Thus, intronal SNPs influence the genetic susceptibility to cancer by both genetic and epigenetic mechanisms (Figure 3).

### Intronal SNPs affect gene expression by cis-acting regulatory elements

Intronal sequences contain many cis-acting regulatory elements such as transcription factors, enhancers, silencers, and insulators that positively regulate gene expression and are common “hot spots” in genetic risk variants [74]. The current focus of research is to understand the functional consequences of these loci.

A GWAS identified rs2981578 variant in fibroblast growth factor receptor 2 (FGFR2) as one of the highest ranking risk alleles in breast cancer. Heterozygous rs2981578 clones showed high levels of FOXA1 transcription factor binding to this intronal SNP [75]. The rs12343867 T > C variant in Janus kinase 2 intron 14 is associated with myeloproliferative neoplasms by acting as a transcriptional repressor [76].

Cowper-Sal lari *et al.* found that breast cancer risk variants targeted enhancers of the FoxA1 and ESR1 transcription factors. The rs4784227 SNP in the *TOX3* gene modulated the affinity of chromatin for FOXA1 in distal regulatory elements resulting in allele-specific gene expression [77]. Three independent variants (rs2981578, rs35054928 and rs45631563) of FGFR2 map to transcriptional silencer elements and augment silencer activity resulting in lower FGFR2 expression and increased estrogen responsiveness and breast cancer risk [78].

### Intronal SNPs regulate protein synthesis by mRNA splicing

The mRNA splicing involves splice donor and acceptor sites, exon splicing enhancers, and splicing proteins. Sequence changes due to non-synonymous or synonymous SNPs modulate mRNA splicing activity resulting in the production of splice variants. For example, SNP resulting from G to A in *DMD* intron 32 deactivates splice donor sites [79]. A polymorphism at a splice donor site in *ZNF419* produces ZAPHIR, an alternatively spliced polymorphic histocompatibility antigen in renal cell carcinoma [80]. An intronal polymorphism in *IRF4* is associated with increased risk of male acute lymphoblastic leukemia, wherein the C to T substitution increases *IRF4* gene expression by transcriptional repression. The wild-type C allele shows two-fold stronger binding to the transcription factor AP-2α than the variant T allele. AP-2α plays a negative regulatory role in some tumor cells, although it is known promote transcription. Wild-type *IRF4* significantly inhibits the *IRF4* promoter, but is reversed by the replacement of C by T [81].

### Intronal SNPs influence genomic imprinting

Genomic imprinting refers to differential expression from maternal and paternal alleles due to variations in DNA methylation and histone acetylation. Polymorphisms in the imprinted regions affect gene expression. *H19* is an imprinted gene that codes for an oncofetal RNA, but is down-regulated postnatally. Heterozygotes for the *H19* rs2839698 TC intronal SNP are protected against bladder cancer, especially non-muscle-invasive bladder cancer. The *H19* RNA inhibits insulin-like growth factor 2 (IGF2) transcription and translation, whereas the *H19* SNP promotes mitotic IGF2 expression, thereby decreaseing the risk of bladder cancer [82].

### Intronal SNPs regulate gene expression via lncRNAs

Intronal sequences contain many non-coding RNA motifs, which do not encode proteins but regulate protein expression through epigenetic, transcriptional and post-transcriptional regulation. As an important member of non-coding RNAs, lncRNAs (long non-coding RNA) are not only involved in chromatin remodelling, histone and chromatin modifications, but also participate in transcriptional activation or repression, alternative splicing, endosome transport and oncogene/tumor suppressor activation or repression. LncRNAs are closely related to malignant tumorigenesis. Polymorphisms in lncRNA-encoding genes affect normal biological functions by competitive microRNAs, thereby influencing susceptibility to cancer. The rs2147578 polymorphism in lncLAMC2-1:1 transcript influences the binding of miR-128-3p and correlates with colorectal cancer (CRC) risk. The CG and GG genotypes of the rs2147578 polymorphism are associated with increased risk for CRC [83]. Numerous genetic polymorphisms in lncRNAs are associated with increased risk in gastric cancer, CRC and prostate cancer [84–86]. SNPs in the lncRNAs H19, HOTAIR (HOX transcript antisense RNA) and PRNCR1 correlate with increased gastrointestinal cancer risk [87]. The SNP rs920778 with T allele increases the expression of lncRNA *HOTAIR* via a novel intron enhancer and is associated with ESCC susceptibility [88]. Many SNPs of *PRNCR1* are associated with prostate cancer susceptibility. *PRNCR1* expression is increased in prostate intraepithelial neoplasia and prostate cancer, whereas tumour cell viability and androgen receptor activity is decreased when *PRNCR1* is silenced [89]. The rs10680577 insertion/deletion polymorphism of *EGLN2* results in high expression of both EGLN2 and RERT-lncRNA and positively correlates with liver cancer risk. It is a potential biomarker for early diagnosis of liver cancer and has been postulated to disrupt the structure of RERT-lncRNA resulting in changes in EGLN2 expression [90]. The NBAT-1 lncRNA inhibits neuroblastoma progression by decreasing cell proliferation and promoting neuronal differentiation [91].

### Intronal SNPs affect chromatin looping

In male-specific childhood ALL, intronal SNP rs12203592, located in intron 4 of the IRF4 gene, enhances physical interaction of the enhancer with the IRF4 promoter through an allele-dependent chromatin loop resulting in higher IRF4 transcriptional rate [92, 93].

## UTR-RELATED SNPs AND CANCER SUSCEPTIBILITY

The 5′ and 3′ UTRs of mRNAs are critical because they control translation [94]. The 5′-UTR regulates translation initiation, whereas the 3′-UTR determines mRNA stability. Specific regulation of mRNA translation is an essential part of gene expression and can be modulated by sequence variations in the 5′ and 3′ UTRs.

Single nucleotide variations (SNVs) are highly disruptive as they change the secondary structure and miRNA target sites within UTRs [95]. These changes alter the expression of known cancer related genes and signaling pathways. Sequence changes in the UTR regions affect mRNA folding that impacts transcript stability, mRNA processing and/or translational control. Thus, UTR-SNPs (non-coding SNPs located in the UTR) may have functional consequences on mRNA stability and/or expression [96].

### SNPs in 5′-UTRs affect transcription and protein translation

The 5′-UTR includes genetic elements that regulate gene expression. It starts at the transcription start site and ends at the nucleotide before the start codon. Polymorphisms in 5′-UTRs have been linked to many human diseases because they regulate mRNA processing, transport, stability, and translation. The overall translation rate is influenced by the length of the 5′-UTR, translational start site and its secondary structure, upstream AUGs, upstream open reading frame (ORF) and ribosome binding sites (internal ribosome entry sites, IRESs) [97]. Therefore, polymorphisms or mutations in the 5′UTR can affect translation efficiency. In mammalian genes, ribosomes bind directly to the IRES of 5′-UTR region [97]. In multiple myeloma, a +2756 C to T mutations in the 5′-UTR of the *c-Myc* gene increase the activity of IRES, thereby promoting *c-Myc* expression and tumorigenesis [98]. In the 5′UTR of *CDKN2A* gene, a -34 G to T substitution results in the addition of a new upstream ORF that inhibits translation, which results in loss of allele function and increases melanoma risk [99].

The 5′-UTR also plays a role in transcription activity. The +24 T/C polymorphism in the 5′-UTR of the *CR2* gene is associated with nasopharyngeal carcinoma risk. Individuals with the minor allele C are at a higher risk for NPC than those carrying the T allele (OR = 1.81) due to increased transcriptional activity [100].

### SNPs in 3′-UTRs regulate mRNA degradation and translation

**Figure 4:**
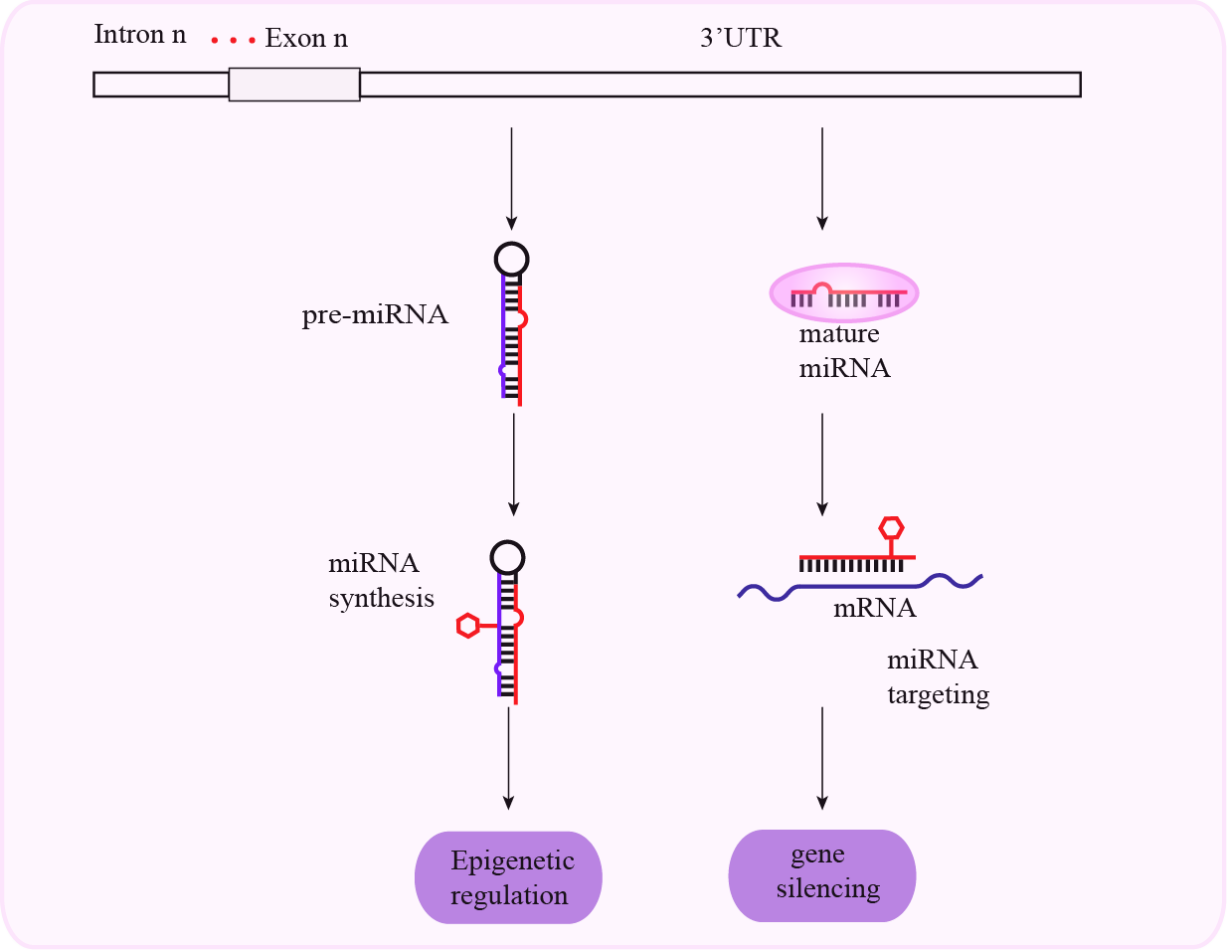
Schematic representation of mechanisms associated with 3′-UTR SNPs and cancer susceptibility SNPs in the 3′-UTR affect miRNA synthesis and gene silencing by altering miRNA-mediated translational repression.

The 3′-UTRs regulate gene expression through mRNA degradation and translation. The 3′-UTR controls polyadenylation, subcellular localization, translation efficiency, and mRNA degradation. It also determines the fate of specific mRNAs and cell type-specific mRNA expression. Therefore, mutations in the 3′-UTR are involved in many diseases because they affect gene progression. As a regulatory region, the 3′-UTR is indispensable for normal gene expression. Therefore, polymorphisms in the 3′-UTR and miRNAs can alter miRNA binding sites and affect mRNA degradation and protein translation (Figure 4).

### SNPs in 3′-UTRs alter miRNA-mediated translational repression

The microRNAs (miRNAs) inhibit translation and destabilize their target mRNAs by binding to the 3′-UTR of the target transcript [101–103]. SNPs in 3′-UTRs alter target recognition of microRNAs by disrupting sequence complementarity. Some polymorphisms interfere with the function of the miRNA and affect the expression of the miRNA targets [104, 105]. The rs93410170 C > T SNP in the 3′-UTR of the estrogen receptor-α (*ER-α*) gene results in stringent miR-206-mediated regulation of *ER-α* expression and is associated with high risk of breast cancer [106]. Polymorphisms or mutations in the 3′ end of target genes affect protein translation even in the absence of changes in mRNA expression. The C allele of rs10889677 A > C polymorphism in the 3′-UTR of *IL23R* is associated with breast cancer, lung cancer, and nasopharyngeal carcinoma in Chinese individuals; the A allele eliminates miR-let-7f binding sites, thereby increasing *IL23R* transcription [107].

SNPs in miRNAs can inhibit miRNA processing and targeting. Mature miRNAs include a 5′-seed region and a 3′-mismatch-tolerant region. The seed sequence is made up of two to seven nucleotides at the 5′ end of the miRNA and is involved in the specific identification of the miRNA targets. Therefore, SNPs in the seed sequence can interfere in the binding of the miRNA to its target mRNA. The rs11614913 T > C SNP in miR-196a2 and rs3746444 A > G SNP in miR-499 are associated with increased breast cancer risk [108]. Polymorphisms or mutations in miRNA alter binding sites, thereby inhibiting gene expression and protein synthesis in the absence of any changes in the encoding mRNA.

### SNPs in miRNAs

The miRNA polymorphisms or mutations outside the pre-miRNA hairpin-shaped structure and the miRNA seed sequence can affect the synthesis of miRNAs. For example, pri-miRNA polymorphisms such as pri-miR-34 b/c rs4938723 [109], pri-miR-218 rs11134527 [110], and pri-miR-938 rs2505901 [111] are used as biomarkers to predict hepatocellular and gastric cancer risk. A polymorphism in the seed region of miR-125a inhibits the processing of the pri-miR-125 to pre-miR-125a, thereby reducing miR-125a mediated translational repression [112]. A C > T polymorphism in the primary transcript of miR-15a/miR-16 is associated with reduced miR-15 and miR-16 expression in familial chronic lymphocytic leukemia [113, 114].

## SNPs IN UNDEFINED GENETIC REGIONS AND CANCER SUSCEPTIBILITY

### SNPs in undefined genetic regions modulate gene transcription through long range cis effects

SNPs in non-coding regions regulate their target genes through long-range chromatin interactions. Most of these interactions are located in sites with active histone modifications and transcription factor binding sites. Many candidate genes, such as CAPG, C2orf43, RFX6, NFASC, MYC and AGAP7P and their regulatory variants, including rs1446669, rs699664, rs1078004, rs13394027, rs10993994 and rs4631830 were identified in prostate cancer, thereby showing the role of long-range chromatin interactions [115]. Multiple functional variants in long-range enhancer elements are associated with SNP rs965513, regulated the expression of FOXE1 and PTCSC2, and contribute to thyroid cancer risk [30]. Lo *et al.* demonstrated that multiple variants co-operated with the lead SNP and long-range enhancers to transcriptionally regulate FOXE1 and PTCSC2 expression [30]. Functional variants at the 11q13 risk locus for breast cancer downregulate cyclin D1 expression through long-range enhancers [116]. A transcriptional enhancer in the G allele of rs554219 increases the risk of breast cancer by reducing the binding of ELK4 transcription factor and correlates with low cyclin D1 levels in breast cancer tissues [116]. The rs6983267 variant in a transcriptional enhancer on chromosome 8q24 is associated with colorectal cancer pathogenesis because it binds differentially to transcription factor 7-like 2 (TCF7L2) and cMyc [117].

### SNPs in tRNAs and rRNAs cause transcriptional and translational defects

In some cases, changes in tRNAs and rRNAs are associated with cancer susceptibility. Mutations in the mitochondrial tRNA genes alter the secondary and tertiary tRNA structure, leading to transcriptional and translational defects in the mitochondrial respiratory chain components. The A12308G is a polymorphic mutation in the V-loop of tRNALeu (CUN), which is associated with colorectal cancer susceptibility [118]. Mutations in the mitochondrial tRNA genes are associated with mitochondrial dysfunction in breast cancer [119]. A cis non-coding rRNA (nc-rRNA), upstream of the 45S rRNA transcriptional start site is implicated in altered rRNA transcription in human lung epithelial cancer cells due to changes in its secondary structure [120]. Mitochondrial SNPs (mtSNPs) in the rRNA and tRNAs, MT-ND2 and haplogroup T are associated with CRC risk in European Americans (OR = 1.66, 95% CI: 1.19–2.33, *P* = 0.003) [121].

## CONCLUSIONS AND PERSPECTIVES

In this systematic review, we describe the role of SNPs in different regions of the gene (promoter, exons, introns and UTRs) in cancer susceptibility. SNPs in the promoter region influence promoter activity, transcription factor binding, DNA methylation and histone modifications. SNPs in the exons modulate gene transcription and translation. SNPs in the introns affect RNA splicing, genomic imprinting, and lncRNAs. The SNPs in 5′-UTR promote translation, whereas SNPs in the 3′-UTR modulate miRNA-dependent gene silencing. Moreover, SNPs in other regulatory regions like the enhancers regulate transcription, and translation as well as protein folding (Table 1).

**Table 1:** Molecular mechanisms of region-based SNP on cancer susceptibility

SNP regions	Possible molecular mechanism	Unclear issues
Promoter	Genetic regulation: promoter activity (TATA box, transcription-factor binding ability)	the interaction between genetic and epigenetic elements
	Epigenetic regulation: DNA methylation, histone modification	effect of SNPs on DNA methylation status
Exons	Non-synonymous cSNPs: coding protein structure and function	detail mechanism at biochemical and cellular level
	Synonymous cSNPs: secondary structure conformation translation dynamics	mechanism of kinetics of translation
Introns	cis-regulatory elements mRNA splicing genomic imprinting lncRNAs chromatin looping	detail functions of cis-regulatory elements and splicing
UTRs	5′-UTRs: protein translation and transcription activity.	how SNPs in the 5′-UTR affect the efficiency of translation
	3′- UTRs : Regulate mRNA degration and translation	how the 3′-UTR affects miRNA binding sites
Non definite regions	long range cis regulation tRNA and rRNA	the ways polymorphisms affect long range cis regulation, tRNA and rRNA

Despite extensive research into the role of SNPs in genetic predisposition of cancer, the mechanisms remain complex. For example, while it's well known that promoter region SNPs affect methylation and histone modifications, the co-operative interaction between genetic and epigenetic mechanisms remains unclear. Moreover, while genetic evidence exists for association between exonal polymorphisms and cancer susceptibility, biochemical and cell biological outcomes of non-synonymous cSNPs are not known. Also, the mechanistic details regarding the effect of synonymous SNPs on the kinetics of translation need detailed investigation. The effect of DNA methylation, which mainly occurs in the promoter region and the first exon, needs to be studied in relation to SNPs. While the functions of intronal polymorphisms are well understood, their effects on splicing, cis-regulatory elements and lncRNAs require further study. Also, the role of polymorphisms in untranslated regions in gene expression through epigenetic regulation of translation has been recognized. However, the effect of SNPs in the 5′- and 3′UTRs in translation, DNA methylation, miRNA binding, and tissue-specific gene expression needs to be further investigated. Polymorphisms in the pri-, pre-, and mature miRNAs influence selection of target miRNAs and affect expression of the numerous proteins and signaling pathways. The mechanisms related to gene polymorphisms and cancer susceptibility is very complex. Gene polymorphisms also change spatial structure, which affects mRNA stability, methylation and allele-specific expression. These mechanisms require further detailed investigation.
